# Synthesis and Characterization of Crosslinked Castor Oil-Based Polyurethane Nanocomposites Based on Novel Silane-Modified Isocyanate and Their Potential Application in Heat Insulating Coating

**DOI:** 10.3390/polym14091880

**Published:** 2022-05-04

**Authors:** Yuan Meng, Ken Chen, Yuyin Yang, Tao Jiang, Tonghui Hao, Xiaoju Lu, Qunchao Zhang

**Affiliations:** 1 School of Materials Science and Engineering, Hubei University, Wuhan 430061, China; 201901111300099@stu.hubu.edu.cn (Y.M.); 202121113012842@stu.hubu.cn (K.C.); 202121113012884@stu.hubu.edu.cn (Y.Y.); jiangtao@hubu.edu.cn (T.J.); haoth@hubu.edu.cn (T.H.); 2 School of Chemistry and Chemical Engineering, Hubei Polytechnic University, Huangshi 435005, China

**Keywords:** silane grafted IPDI tripolymer, castor oil, bio-based polyurethane, heat-insulating coating, hybrid materials

## Abstract

An isocyanate with trimethoxysilane groups at the side chains (IPDI-M) was synthesized via an addition between the mercaptopropyl trimethoxysilane groups (MPTMS) and IPDI tripolymer (IPDI-T). Then, silane grafted isocyanate as the functional hard segment, castor oil as the soft segment, poly (ethylene adipate) diol (PEA) as the chain extender, and MPTMS as an end-capping reagent were applied to form a series of organosilicon hybrid bio-based polyurethane (CPUSi). The effect of the IPDI-M contents on the thermal stability, mechanical properties, and surface properties of the resulting product was systematically investigated. Profit from the Si–O–Si crosslinked structures formed from MPTMS curing, the tensile strength, and Young’s modulus of the resulting products increased from 9.5 MPa to 22.3 Mpa and 4.05 Mpa to 81.59 Mpa, respectively, whereas the elongation at break decreased from 342% to 101%. The glass transition temperature, thermal stability, transparency, hydrophobicity, and chemical resistance were remarkably strengthened for the obtained organosilicon-modified polyurethane with the increasing MPTMS content. At the end of the work, the thermal insulation coating that was based on CPUSi and ATO can effectively block near-infrared rays, and the temperature difference between the inside and outside of the film reached 15.1 °C.

## 1. Introduction

With the increasing global demand for energy, energy consumption also brings environmental problems, therefore, energy and the environment have become a bottleneck, restricting the development of human society. Building energy consumption accounts for about 30% to 40% of the total human energy consumption, nearly half of which is caused by air conditioners, such as making heating or cooling, while the heat lost through doors and windows accounts for about 30% of the energy consumption of air conditioning in the whole building. On the other hand, with the improvement of modern buildings’ requirements for outdoor landscape and indoor lighting, large glass windows, or glass curtain walls are often used, and building glass is the weak link in thermal insulation. How to improve the performance of thermal insulation while ensuring glass lighting has become a meaningful way to reduce building energy consumption.

In recent years, with the gradual maturity of nanomaterials and preparation technology, some metal oxides, such as indium tin oxide (ITO) [1], tin-antimony oxide (ATO) [2,3,4], and aluminum-doped zinc oxide (AZO) [5], have attracted more and more attention in the field of thermal insulation fillers because of their unique optical and thermal properties. When these nanomaterials are used in glass coating and spraying, choosing a suitable polymer substrate is necessary. In particular, when the transparent thermal insulation coating is used in the layer of building glass, it needs to withstand solar radiation and exert its fine and thermal insulation function. In contrast, the resin in the transparent thermal insulation coating ages easily when exposed to solar radiation for a long time. At the same time, the thermal insulation coating used on the outside of building glass often experiences atomization or even blistering and delamination because of rainwater; therefore, it is necessary to improve the aging resistance and water resistance of transparent thermal insulation coatings. The thermal insulation coating is mainly composed of thermal insulation filler and resin, and the resin acts as a bridge between the thermal insulation filler and the glass substrate. Therefore, to prolong the service life of transparent thermal insulation coating and improve its energy-saving performance, in the final analysis, it is necessary to improve the comprehensive properties, such as waterproofing and weather resistance of resin.

Polyurethane (PU) has become the leader among many candidate resins because of its excellent properties (such as high weather ability, adjustable surface hardness (soft and hard)), good chemical resistance, and elevated glass transition temperature (Tg), and formula adaptability [6,7,8]. What is more pleasant, however, is that the research and development and application of bio-based polyurethane are constantly advancing [9,10,11], which is undoubtedly a favorable trend to weaken the consumption of fossil fuel resources, reduce greenhouse gas emissions and protect the environment. However, the comprehensive performance of bio-based polyurethane is still slightly inferior to that of traditional polyurethane, therefore, structural modification and hybrid materials have become a powerful means to improve the all-around performance of bio-based polyurethane [12,13,14,15]. Researchers have effectively enhanced bio-based polyurethane’s mechanical properties and thermal stability by compounding polyols [16], introducing rigid groups into the structure [12], adjusting the degree of crosslinking [14], etc. and further hybridizing with inorganic materials [17,18,19,20,21,22,23,24,25,26] to endow bio-based polyurethane with electrical conductivity, thermal conductivity, and other functions. According to the previous research results, reducing the crosslinking density of bio-polyurethane is beneficial for enhancing flexibility; however, it also leads to a decrease in strength and modulus, and although the introduction of rigid groups into the trunk is helpful to increase the strength and modulus, the improvement in flexibility is limited. By contrast, organic–inorganic hybrid bio-based polyurethane has more controllable properties, which could significantly improve the different properties of bio-based polyurethane according to the type of nanofillers. However, as far as thermal insulation coating is concerned, nanofiller can only choose nanoparticles with thermal insulation effects, such as ATO, GTO, etc. Therefore, the previous research which focused on organic–inorganic hybrid thermal insulation coating is more inclined to study the compatibility between thermal insulation filler and organic polymer and rarely involves improving water resistance and weather resistance. As we all know, a silane coupling agent is an excellent choice to improve the compatibility between two surfaces. In fact, the polyurethane with silicone alkyl group in its structure has more obvious performance advantages, mainly since organosilane is hydrolyzed and condensed to form an organic silsesquioxane crosslinked structure during the film-forming process. Improving the crosslinking degree and developing a silicone/polyurethane hybrid structure is beneficial to enhance its water and weather resistance. More interestingly, silicone alkyl groups can quickly form hydrogen bonds or condense into covalent bonds with hydroxyl-containing surfaces during hydrolysis, which can undoubtedly improve the adhesion. To sum up, for the bio-based polyurethane used in the field of thermal insulation, the introduction of a silicone alkyl group into the polyurethane structure cannot only enhance the water resistance and weather resistance of the thermal insulation coating but also help to enhance the adhesion between filler and polyurethane, glass and polyurethane, and prolong the service life of the thermal insulation coating. Many researchers have employed 3-aminopropyltrimethoxysilane (APTMS) to generate silicified polyurethane hybrid materials with excellent properties [21,27,28]. However, in that case, silicon-containing groups can only be located at both ends of the polyurethane prepolymers, limiting the content of silane groups that can be incorporated into the polymer chain, and, thus, is not conducive to regulating the distribution of silane groups as well. Another method is to graft silane groups on the side chain of polyurethane. At present, a few studies have reported that the introduction of silane groups into polyurethane side chains by silane as a chain extender can effectively improve the mechanical, heat, and water resistance of polyurethane [29,30,31]. Based on the above analysis, if silane can be introduced from the soft segment or hard segment of polyurethane, it may also be beneficial to improve the mechanical properties, heat resistance, and water resistance of polyurethane by adjusting the distribution and content of silane groups. To date, compared with the soft segment grafted silane coupling agents which has been reported in the literature [32], the work on the design and synthesis of hard segment grafted silane coupling agents is rarely reported.

**Scheme 1:**
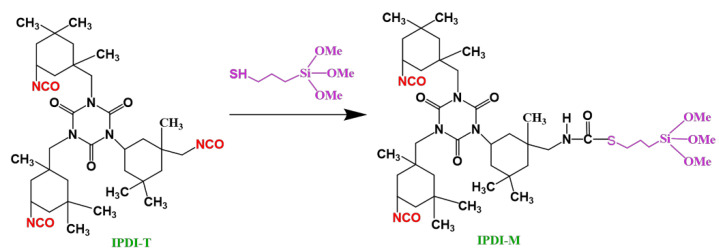
Synthesis of IPDI-M.

**Scheme 2:**
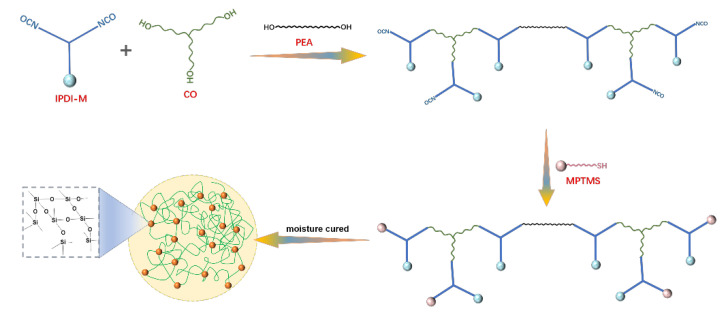
Synthetic route of CPUSi.

In this contribution, a new strategy was used to modify castor oil-based polyurethane with silane grafted hard segment to improve the mechanical properties, heat resistance, solvent resistance, and adhesion. Firstly, mercaptopropyl trimethoxysilane (MPTMS) containing one mercapto group and three methoxysilyl groups reacted with isocyanate trimer (IPDI-T) to obtain a new type of isocyanate with silane side group (IPDI-M) (Scheme 1). Further, a novel silicone hybrid bio-based polyurethane was synthesized by IPDI-M, CO, PEA, and MPTMS (Scheme 2). In particular, IPDI-M with the silane side group as a functional hard segment is beneficial to enhance the crosslinking degree and form a polysiloxane structure during the moisture-curing process, while CO as biological polyols is employed to prepare bio-based polyurethane. In addition, the capping of the end of the chain with MPTMS is conducive to eliminating the residual toxic isocyanate groups in the structure, further increasing the silane content in the polyurethane structure and reducing damage to the human body and environment during the use of the product. The effects of different IPDI-M contents on the CPU were studied by using Fourier transform infrared spectroscopy (FTIR), an X-ray diffractometer (XRD), X-ray photoelectron spectroscopy (XPS), scanning electron microscopy (SEM), an atomic force microscope (AFM), a differential scanning calorimeter (DSC) and thermal gravimetric analyzer (TGA). Meanwhile, the optical performance, mechanical property, adhesive property, surface properties, and chemical resistance properties of the CPU with different IPDI-M contents were evaluated in our present work. Finally, the new bio-based polyurethane was compounded with different contents of ATO, and its thermal insulation property was measured.

## 2. Materials and Methods

### 2.1. Materials

3-isocyanatomethyl-3,5,5-trimethylcyclohexyl isocyanate (IPDI) and IPDI tripolymer(IPDI-T) were purchased from Bayer, while dibutyltin dilaurate (DBTDL) and triethylamine were bought from China Sinopharm Group, Shanghai, China. They were used as received. The castor oil (CO) from China Sinopharm Group, Shanghai and poly (ethylene adipate) diol (PEA, Mw = 600 g/mol) from Jining Huakai Resin Co. Ltd., Jining, China, were dried at 110°C under reduced pressure for 3 h before use. Butanone was obtained from China Sinopharm Group and was refluxed over CaH2 and distilled before use. Mercaptopropyl trimethoxysilane (MPTMS) was kindly supplied by Hubei New Blue Sky Co. Ltd., Xiantao, China and was used without processing.

### 2.2. Preparation of MPTMS Grafted IPDI-Tri (IPDI-M)

As presented in Scheme 1, MPTMS and IPDI-Tri with three drops of triethylamine were added to the 250 mL four-neck round-bottom flask full of argon. Experiments were performed at 75 °C until the content of the isocyanate groups reached a theoretical target value Here, the theoretical target value was calculated by the following equation and the actual measured value was acquired by titration with the classic di-n-butylamine method (HG/T 2409-1992). Then, the target IPDI-M was obtained.

$$NCO\left(wt\%\right)=\frac{\left(n_{–NCO}-n_{–SH}\right)}{m}\times 42\times 100\%$$

In the formula: n_–NCO_ and n_–SH_ represent the amount of substance of the NCO group and the SH group, respectively; m represents the total mass.

### 2.3. Preparation of Silicone/Castor-Based Polyurethane (CPUSi) Hybrids Coating

CPUSi was synthesized by the two-step polymerization procedure, as presented in Scheme 2. In the first place, IPDI and IPDI-M reacted with CO at 75 °C under an Ar atmosphere to prepare CO-based polyurethane precursors until the residual isocyanate groups reached the theoretical value (the following Equation). In the second step, NCO-terminated CPUSi-prepolymer was generated by reacting the precursor with PEA until the residual isocyanate groups obtained the theoretical value, Equation (2). Finally, a homogeneous and transparent CPUSi hybrid solution was procured by blocking with MPTMS at 40 °C until the isocyanate groups were depleted. Then, the CPUSi solution was cast in a tetrafluoroethylene mold and dried at room temperature for a week to obtain the hybrid coatings. Table 1 reveals the amount of raw material composition employed for the preparation of CPUSi.

$$NCO\left(wt\%\right)=\frac{\left(n_{–NCO}-n_{–OH}\right)}{m}\times 42\times 100\%$$

In the formula: n_–NCO_ and n_–SH_ represent the amount of substance of the NCO group and the OH group, respectively; m represents the total mass.

### 2.4. Preparation of CPUSi/ATO Hybrids Coating

After the ATO was dispersed in the ball mill for 8 h, it was added to the CPUSi20 according to 1 wt%, 5 wt%, and 10 wt%, and then dispersed to the uniform slurry (ATO/CPUSi) by a high-pressure homogenizer.

### 2.5. Characterization

UV spectrophotometer analysis was performed using a UV-3600 spectrophotometer from 300 to 800 wavelengths. A Fourier transform infrared (FTIR) analysis was measured with a Spectrum One FTIR spectrometer (Nicolet iS50, Thermo Fisher Scientific, Chicago, IL, USA). The film was directly tested on ATR, a liquid sample was directly coated on the dried KBr flakes. A thermogravimetric analyzer (TGA) was performed from 30 to 800 °C under nitrogen conditions at a 10 °C/min ramping rate by using the TG/DTG (Diamond, PerkinElmer, Singapore). The scanning electron microscopy (SEM, JSM-2010) of the films was enforced to observe the surface morphology. Atomic force microscopy (AFM, Prima, NT-MDT Spectrum Instruments Group Company, Zelenograd, Russia) was performed to analyze the surface roughness parameters and the topography of the coatings. The mechanical properties, including tensile strength, Young’s modulus, and elongation at break of synthesized samples, were measured on a CMT4303 universal test machine (SANS, MTS Systems Corporation, Shenzhen, China) at 25 °C with the speed of 100 mm/min, as mentioned on GB/T 13022-91. The liquid NCO-terminated CPU prepolymers were tested after curing in a polytetrafluoroethylene plate with a width of 6.0 mm, a thickness of 0.8 mm, and a length of 25 mm. For the sake of accuracy, take the average of five replicates for each sample. The hydrophilicity of the CPU surface was tested at 25 ± 2 °C in a contact angle instrument (JC2000D1, Shanghai Zhongchen Digital Technology equipment Co., Ltd., Shanghai, China), and every sample was measured five times for averaging.

Swelling ratio and chemical resistance: the dried CPUs recorded as w_1_ were soaked in 20 mL of solvent for 7 days, including deionized water, ethyl acetate, acetone, dichloromethane, dimethyl formamide, 5 wt% H_2_SO_4_, and 5% NaOH. Then, they were taken out to weigh by wiping the surface solvent, recorded as w_2_. Next, the above samples were placed into the vacuum drying oven overnight at 60 °C, and the weight was recorded as w_3_. Finally, the swelling degree and the mass loss ratio were calculated according to the following equations

$$w_{swelling}=\frac{\left(w_{2}-w_{1}\right)}{w_{1}}$$

$$w_{lost}=\frac{\left(w_{1}-w_{3}\right)}{w_{1}}$$

w_swelling_: swelling ratio, w_1_: initial mass of the CPU (g), w_2_: mass after swelling (g), w_3_: mass after degradation (g).

**Figure 1:**
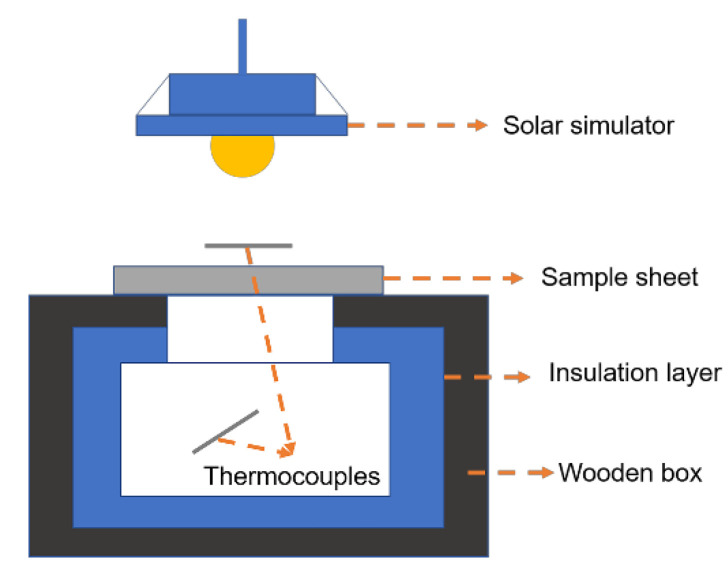
Self-made blackboard thermometer.

Heat insulation properties: a self-made blackboard thermometer was used for testing the heat insulation properties, as shown in Figure 1.

## 3. Results

### 3.1. The Structural Characterization of CPUSi Coatings

#### 3.1.1. Infrared Spectroscopy Analysis

**Figure 2:**
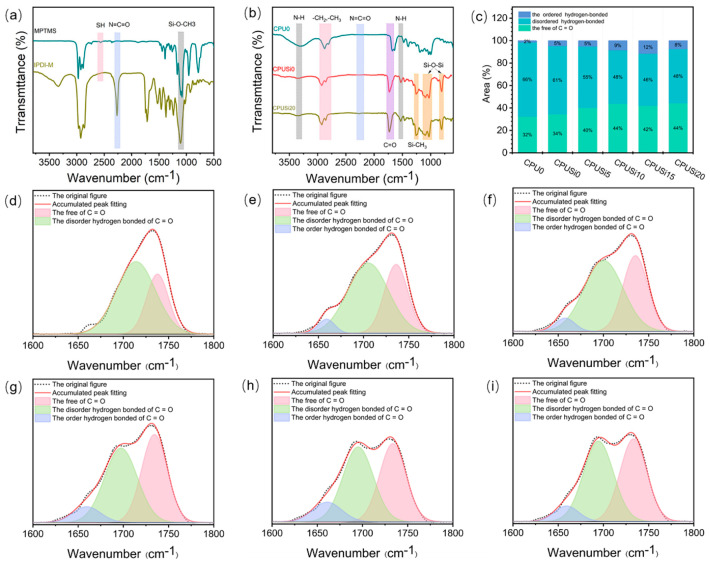
(**a**) FTIR spectra of MPTMS and IPDI-M; (**b**) FTIR spectra of the CPU0, CPUSi0, and CPUSi20 films; (**c**) Comparison of curve-fitting results of the C=O stretching region in CPU and CPUSi hybrids films; (**d**–**i**) Curve-fitting results of CPU0, CPUSi0, CPUSi5, CPUSi10, CPUSi15, and CPUSi20 at the wavenumber range of 1600–1800 cm^−1^, respectively.

The FTIR spectra of MPTMS and IPDI-M are presented in Figure 2a, respectively. As demonstrated in Figure 2a, for IPDI-M, the corresponding peak at 2572 cm^−1^; the vibration of –SH [33] vanished after the N=C=O group was grafted. Meanwhile, a new peak around 1107 cm^−1^ emerges, which is typical for Si–O–CH_3_ compounds [33,34,35], indicating MPTMS as being successfully grafted onto the IPDI tripolymer.

Figure 2b displays a series of CPUSi hybrids films containing different IPDI-M contents (CPUSi0~CPUSi20) and a control film without silane (CPU0). Among them, CPUSi0 refers to the sample that was not added to IPDI-M but was only capped with MPTMS. From 1500 cm^−1^ to 3800 cm^−1^, the curve of the CPU0 film and other CPUSi films presents no obvious difference. The peak at 2270 cm^−1^ (N=C=O stretching) has disappeared, confirming that CPUSis are synthesized successfully [21]. The weak absorption band around 3324 cm^−1^ corresponds to –NH stretching, while 2921 cm^−1^ and 2852 cm^−1^ are attributed to the –CH_2_ and –CH_3_ stretching vibration [29]. The absorption peak at 1730 cm^−1^ signified the formation of C=O stretching of urethane and ester groups [36]. From 600 cm^−1^ to 1500 cm^−1^, the curve of the CPU0 film and other CPUSi films are obviously different. The symmetric bending at 1259 cm^−1^ corresponds to –CH_3_ in Si–CH_3_ symmetric bending [31]. Moreover, the characteristic signal at 1093 cm^−1^ and 800 cm^−1^ are attributable to Si–O–Si asymmetric stretching vibration [31], demonstrating the presence of the siloxane structure. Additionally, the absorption intensity of the absorption peak around 1093 cm^−1^ was slightly stronger with the addition of IPDI-M. The above changes in these groups confirm that the siloxane groups were successfully introduced into the CPU-Si films.

Furthermore, more information on hydrogen bond content and phase separation behavior was obtained by following the change of urethane peaks, where the urethane N–H serves as a proton donor. In contrast, urethane carbonyl oxygen (C=O) and ester oxygen (O−C=O) in the PEA segments serve as proton acceptors [36]. For the basis of this, for investigating the hydrogen bond in polyurethane, the carbonyl region in the 1600–1800 cm^−1^ band was usually decomposed into three peaks at 1650, 1700, and 1733 cm^−1^, which were assigned to the ordered hydrogen-bonded carbonyl, the disordered hydrogen-bonded carbonyl free, and the free carbonyl in turn [36] (Figure 2d–i). The percentage of the hydrogen bonding content could be calculated by the ratio of the hydrogen bonding area to the sum area. As presented in Figure 2c, the hydrogen-bonded ratio of C=O in the five CPUSis with MPTMS decreased from 66% to 56% with the increase of MPTMS content, and they are inferior to that of CPU0 (about 68%). It demonstrates that the MPTMS addition results in the dissociation of hydrogen bonds between –NH and C=O that exist in the CPU. The possible reason may be that the crosslinking network formed by MPTMS hydrolysis interrupts crystallization or chain movement.

#### 3.1.2. X-ray Diffraction Analysis

**Figure 3:**
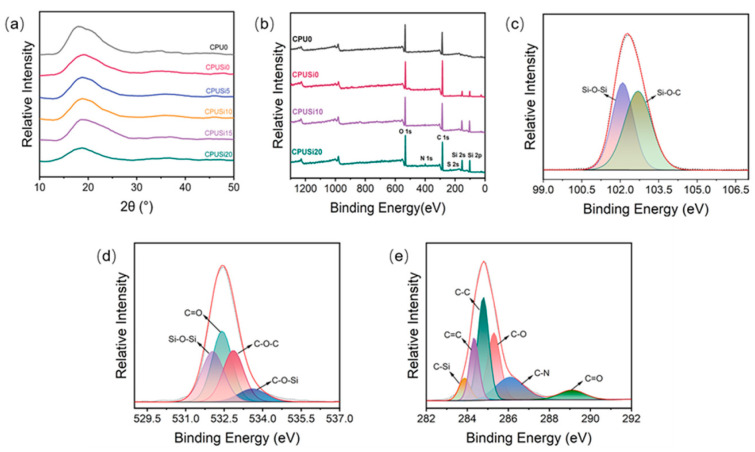
(**a**) X-ray diffractograms of different CPU and CPUSi films; (**b**) XPS spectrum of the film surface of CPU0, CPUSi0, CPUSi10, and CPUSi20 films; (**c**) XPS spectra of Si 2p of CPUSi20 film; (**d**) XPS spectra of O 1s of CPUSi20 film; (**e**) XPS spectra of C1s of CPUSi20 film.

Figure 3a depicts the XRD curves of the CPU and CPUSi samples. Obviously, the series of CPUSi reveal the same characteristic solid peak at 19°, which is similar to that of the pure CPU. These diffraction peaks suggest that no apparent crystallinity survives in any film. In other words, the obtained CPU and CPUSi were amorphous polymers [30]. In addition, compared with the pure CPU, the intensity of the diffraction peaks becomes gradually weak and broad as the IPDI-M content increases, manifesting this crystallinity is lower little by little. As we all know, the diffraction peak is associated with the soft segments in polyurethane. The weakening and widening of the diffraction peak suggest that the regularity of soft segments is decreased. The reason is, therefore, that the Si–O–Si crosslinked network structure, which results from the hydrolysis and condensation reaction of alkoxysilane in IPDI-M and MPTMS, restricted the movement and ordered arrangement of the soft segments [30,37].

#### 3.1.3. XPS Analysis

Furthermore, the XPS survey spectrum investigated the surface chemical elemental composition of CPU0 and CPUSi. As shown in Figure 3b, the signals of carbon (C1s), nitrogen (N1s), oxygen (O1s), and sulfur (S2s) were retrieved at about 284 eV, 399 eV, 530 eV, and 163 eV, respectively. What is worth noting, however, is that additional signals of silicon, including Si 2p and Si 2s, appear separately at 103 eV and 154 eV, proving that the Si element had been successfully introduced into the CPU chain [35]. Moreover, the XPS spectra of Si 2p on the surface of sample CPUSi20 were presented in Figure 3c. The spectrum of Si 2p can be split into two peaks. The binding energies of Si–O–Si and Si–O–C correspond to 102 eV and 102.7 eV, respectively. The finding confirms the formation of the Si–O–Si network [38]. Table 2 list the surface Si elemental concentrations of CPUSi, especially containing the theoretical and experimental value. As we can see, the experimental Si concentration increased from 6.43% (CPUSi0) to 15.25% (CPUSi20) with the increasing IPDI-M content, which was a tower over the theoretical concentration of 1.12% (CPUSi0) to 1.7% (CPUSi20). The phenomenon was relative to the silicon atoms in the crosslinked Si–O–Si structure with low surface energy which are likely to migrate to the surface during the film-forming process [30,37]. Meanwhile, the Si content on the surface obtained in this study was much higher than in our team’s previous work [39], manifesting the availability of the strategy by incorporating the MPTMS from IPDI-M.

#### 3.1.4. DSC Analysis

**Figure 4:**
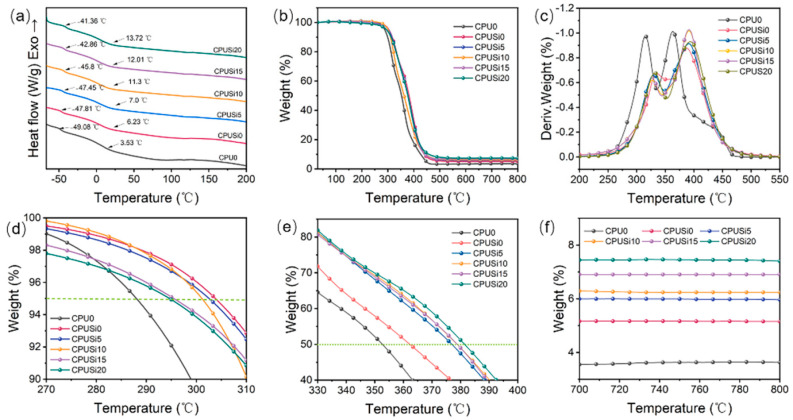
(**a**) DSC curve of CPU and CPUSi hybrids films; (**b**) TGA curve of CPU and CPUSi hybrids films; (**c**) DTG curve of CPU and CPUSi hybrids films; (**d**) TGA curve at 270–310 °C; (**e**) TGA curve at 330–400 °C; (**f**) Char residue at 800 °C.

Figure 4a displays the DSC thermal analysis curves of the CPU and CPUSi nanocomposites. The glass transition temperature (Tg) of CPU0 is −49.08 °C and 3.53 °C, corresponding to the soft segment from CO and PFA, respectively. In contrast, the series of CPUSi had higher Tg with increasing MPTMS contents. In detail, the Tg values of CPUSi0, CPUSi5, CPUSi10, CPUSi15, and CPUSi20 corresponding to CO add up to −47.81, −47.45, −45.8, −42.86, and −41.36 °C, respectively, while the ones corresponding to PFA increase to 6.23, 7.0, 11.3, 12.01, and 13.72 °C, accordingly. The higher shifting of the Tg is related to the increasing crosslink density as well as the dispersion of MPTMS in the CPU matrix, which in turn reduces the soft segmental mobility of the CPU network [21]. Meanwhile, there is no obvious melting or crystallization transition from −70 °C to 200 °C, illustrating chain segments containing low crystallinity as the same as discussed earlier in XRD.

#### 3.1.5. TGA Analysis

**Figure 5:**
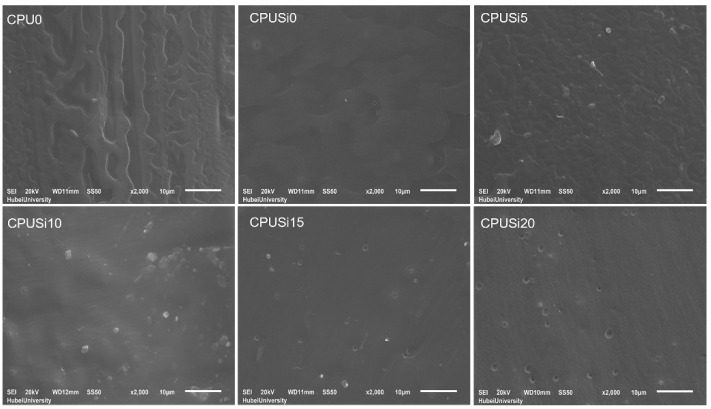
The morphologies of sections in CPU and CPUSi hybrids films.

TGA measured the thermal stability of the CPU and CPUSi composite coating, and the relevant results are summarized in Figure 4b–f and Table 3. T_d5_, T_d50_, and T_max_ refer to the temperatures at which the copolymer undergoes a weight loss of 5 wt%, 50 wt%, and the maximum weight loss rate, respectively. As shown in Figure 4a,b,c, there is a two-stage thermal degradation process in each sample. The first decomposition stage, approximately at the range of 330 °C, was attributed to the decomposition of urethane bonds in the hard segment. The secondary decomposition proceeds in the soft segment at around 396°C, mainly due to the loss of castor oil and PEA [40]. Compared with CPU0, the temperature curves at the maximum thermal mass loss rate of CPUSi moved to the right at each stage (Figure 4c), and, therefore, can be speculated that more crosslinking structure bound the polymer backbones and prevented the polymer chain from moving to increase the thermal stability [21,36]. Furthermore, as shown in Figure 4d, T_d5_ means the initial decomposition temperatures were at 288.2 °C (CPU), 304.1 °C (CPUSi0), 302 °C (CPUSi5), 301.2 °C (CPUSi10), 296.3 °C (CPUSi15), and 294.8 °C (CPUSi20) in turn. What calls for special attention is that the introduction of IPDI-M reduces the T_d5_ values because the average bond energy of C–S (272 kJ/mol) is less than that of C–O (326 kJ/mol) and C–C (332 kJ/mol), resulting in that the initial decomposition temperature gradually decreases with the increase of the IPDI-M content. In contrast, as shown in Figure 5e and Figure 4f, T_d50_ and the residual rate increased from 352.7 to 381.5 °C and 3.6wt% to 7.4wt% individually with the addition of MPTMS content, showing increased thermal stability, which benefits from the forming Si–O–Si crosslinked network and the good barrier effect for heat and mass transfer of the siloxane units [21,40,41]. In a word, the incorporation of IPDI-M can improve the thermal stability of modified castor oil-based PU coatings.

#### 3.1.6. SEM Analysis

Figure 5 reveals the morphologies of the sections of all samples by SEM. The section of the CPU0 is uneven, which is caused by the obvious microphase separation between the soft and hard segments of polyurethane. With the addition of MPTMS, the white spots appeared, which might be siloxane particles [31,41], and the degree of microphase separation became weaker. Further, the white spots increased, and the film’s sections became smoother with the increase of MPTMS. It is well known that the compatibility between soft and hard segments is one of the factors affecting the microstructure of PU. In our study, the hard segments consisted of urethane groups, while the soft ones included castor oil, polyester, and Si–O–Si chains. As seen with the FTIR analysis result, the dissociation of hydrogen bonds accelerated with the increase of MPTMS content, leading to a weakening of the degree of microphase separation. At this point, the microscopic morphology is expressed as smoother and smoother. Moreover, XPS confirms the existence of the Si–O–Si crosslinked network. The SEM shows that more Si–O–Si crosslinked networks may form siloxane particles, which mixes in the polymer matrix as an inorganic strengthening phase of polyurethane.

#### 3.1.7. AFM Analysis

**Figure 6:**
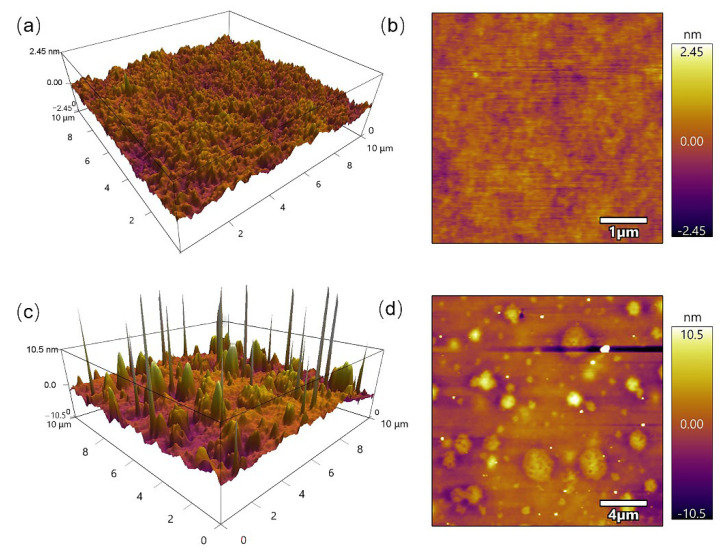
The AFM images of CPU and CPUSi hybrids films (**a**) and (**c**) are 3D maps of CPU0 and CPUSi20, while (**b**) and (**d**) are 2D maps of CPU0 and CPUSi20, respectively.)

The surface roughness of the CPUSi samples can be analyzed by tapping the mode of AFM. Figure 6a,b show 3D and 2D AFM images of the CPU0 coating surface, respectively, while Figure 6c,d present those of the CPUSi20 surface, respectively. Obviously, the surface of CPU0 film is flat (Ra = 223.06 pm). On the other hand, there were many projections and depressions on the surface of the CPUSi20 film (Ra = 1.69 nm). The local bulge is inferred to belong to siloxane particles according to the SEM diagram. In general, the AFM images confirmed that the siloxane particles formed by the hydrolytic crosslinking of silane introduced by IPDI-M were mixed into the polymer matrix to form organic–inorganic hybrid materials.

### 3.2. The Performance of CPUSi Coatings

#### 3.2.1. Optical Performance

**Figure 7:**
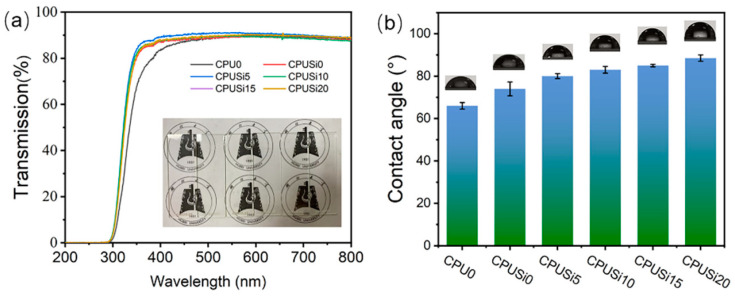
(**a**) The transmittance; (**b**) Water contact angle of CPU and CPUSi hybrids films.

Many studies have shown that silicone-modified PU coatings present high transmittance, which is very suitable for particular purposes, such as optical devices and windscreens. As demonstrated in Figure 7a, in the case of glass slides as a background control, the transmittances of samples are close to 90%, especially the transmittances of the layer prepared with IPDI-M, which have basically no change. As is well known, the transparency of the coatings is affected by many factors, such as the thickness, crosslinking density, and film-forming conditions [20,42]. After coating the CPUSi, just as the XRD test results show that the CPUSi is an amorphous block copolymer, the internal molecules are disordered and are non-crystalline or low-crystalline. When the beam passes through the CPUSi coating, the scattering will not occur, thus the transmittance will almost not change before and after the coating. The value of concern, however, is that the transparency of the layers in our work is significantly higher than in previous reports [12]. Therefore, excellent transmittance maybe suggests that the coating has tremendous application potential in transparent coatings.

#### 3.2.2. Surface Property

To confirm the impact of MPTMS on the water resistance of coatings, the water contact angles (WCA) were tested on the all-samples surface, as shown in Figure 7b. The average contact angles of CPU0, CPUSi0, CPUSi5, CPUSi10, CPUSi15, and CPUSi20 were 66°, 74°, 80°, 83°, 85°, and 88.5°, respectively. Obviously, the water contact angle was increased due to the addition of MPTMS. It has been reported that the crosslinking structure and low surface tension could increase hydrophobic properties [43]. Hence, due to low surface tension and a high degree of crosslinking of the Si–O–Si network structure, which is formed by introducing MPTMS, the contact angles of the CPUSi coating are enlarged, and water molecules are harder to moisten on the surface of the layer.

#### 3.2.3. Chemical Resistance Property

For the purpose of comparing the chemical resistance and water resistance of the films, the films were immersed in different chemical solutions, including ethyl acetate, acetone, dichloromethane, dimethylformamide, 5 wt% H_2_SO_4_, 5 wt % NaOH, and deionized water, for a given period before their performance was evaluated according to the weight variations before and after soaking.

**Figure 8:**
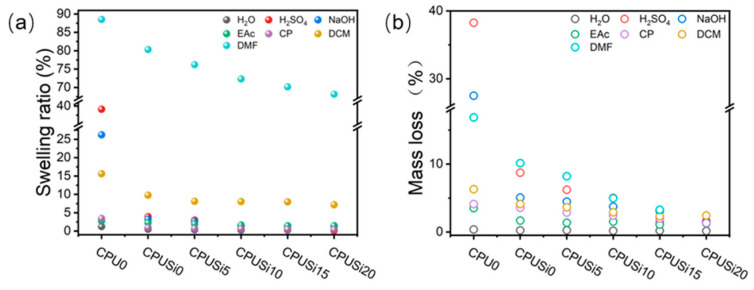
(**a**)The swelling ratio; (**b**) The mass loss of CPU and CPUSi hybrids films.

The chemical resistance property is essential for the weatherability of coatings. The permeability of the medium in the polymer determines the material’s corrosion resistance. The greater the permeability of the medium to the material, the worse the material’s corrosion resistance. From the previous discussion, CPU and CPUSi are amorphous polymers. The solvent molecules are easy to infiltrate into the polymer materials because of the relatively loose aggregation of polymers and rather large molecular gap. As noted in Figure 8a, it is interesting that the swelling ratio of CPU0 is largely increased in DMF but almost unaffected in water, EAc, and CP. Low absorption of CPU0 in water (about 1.24%) is due to the crosslinking structure, implying that castor oil-based polyurethane has excellent water repellent potential. High absorption of CPU0 in DMF (approximately 88.55%) in CPU0 is signifying whose solubility parameter is close to DMF. Meanwhile, it also shows that DMF may corrode CPU0 most. In addition, the solvent absorption of CPU0 in H_2_SO_4_ and NaOH is 39.12% and 26.23%, which is related to the aggravation of hydrolysis of the ester bond under acidic or alkaline conditions. As reported, the swelling largely relies on crosslink density [44,45,46]. In our work, with the addition of MPTMS, the increase in the Si–O–Si crosslinking density and the decrease of the free volume of the molecular chain led to the rise in the average free path of the solvent, so that the solvent swelling ratio was low to 0.12% (H_2_O), 0.13% (H_2_SO4), 1.28% (NaOH), 1.47% (EAc), 0.42% (CP), 7.19% (DCP), 68.16% (DMF), respectively.

To verify the loss rate of the CPU and CPUSi in different solvents, the weight loss value is calculated and then drawn in Figure 8b. The weight loss of CPUSi is smaller than that of CPU0, indicating that the addition of MPTMS does improve the chemical resistance of the material. More importantly, the maximum weight loss of CPUSi20 occurs in DMF, which is only 2.52%. Conclusions can be drawn from the above data that the introduction of organosilicon can achieve strong corrosion resistance by enhancing the barrier to solvents in the high-end application of composite coatings.

#### 3.2.4. Mechanical Performance

**Figure 9:**
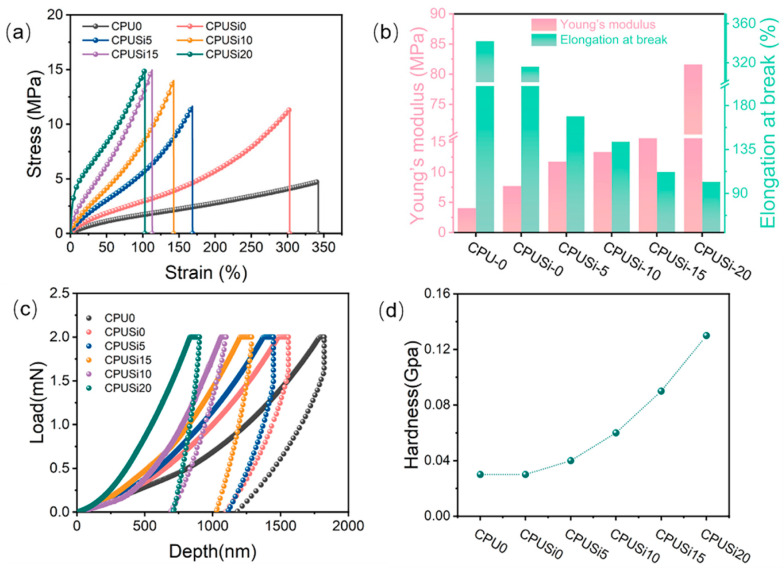
(**a**) Strain–stress curves; (**b**) Tensile properties; (**c**) Load-displacement curves; (**d**) Hardness values of CPU and CPUSi hybrids films.

Figure 9a exhibits the stress–strain curves of the CPU with various MPTEM incorporations. Meanwhile, their tensile strength and Young’s modulus are shown in Figure 9b. The CPU with soft and strict mechanical performance shows an elongation at a break of 342%, the tensile strength of 4.72 MPa, and Young’s modulus of 4.05 MPa. CO contains flexible chains with low molecular weight, and the flexible chains of PFA further increase the chain lengths between the crosslinking sites. Thus, the flexible structure results in low strength and favorable elongation at the break of the material, which accounts for the CPU’s soft and rigid mechanical properties. After MPTEM is incorporated into the polyurethane system incrementally, the tensile strength and Young’s modulus increase, but the elongation at break decreases, benefitting from the further self-crosslinking curing in the later film formation of MPTEM. Comparing CPUSi0 with CPUSi5, the tensile strength, and elongation at the break of the former are 11.3 MPa and 302%, respectively, while the latter is 11.6 MPa and 168%. Obviously, adding a small amount of IPDI-M significantly reduces elongation at break, but little improves the tensile strength.

Further, when adding more amounts of IPDI-M, the tensile properties and elongation at break showed changes sharply at the same time. Compared with CPUSi0, the elongation at break of CPUSi15 dropped to 112%, which is about a one-third times as much as that of CPUSi0, but tensile properties and Young’s modulus raised from 4.72 to 14.89 MPa and 7.71 to 18.25 MPa, which approximately increase 3.15 times and 2.3 times. However, for CPUSi20, which performs more rigidity, the elongation at break dropped to 101%, and tensile properties raised to 14.9 MPa; both are close to that of CPUSi15. Significantly, Young’s modulus of CPUSi20 even grew to 81.59 MPa, which is much stronger than that of CPUSi15. The reason was that the ultra-high crosslinked network formed by Si–O–Si was densely packed in the polymer matrix. The polymer chain was severely bound, leading to enhanced deformation resistance.

Figure 9c displays the surface hardness of the CPU with various MPTEM incorporations obtained from the nanoindentation tests. The depth of CPUs is observed to be shallower with the increasing of the MPTEM contents when all samples load at 2 mN. Specifically, the hardness values of CPUSi20, CPUSi15, CPUSi10, CPUSi5, CPUSi0, and CPU0 are 0.13, 0.09, 0.06, 0.04, 0.03, 0.03 GPa as presented in Figure 6d. Obviously, the hardness value of the first one is nearly four times as much as that of the last one. It is no doubt that the hardness increases gradually with the increase of MPTEM. It is well known that the hardness is related strongly to the micro-nano structure in the polymer network and the bonding between the atoms [47,48,49,50,51]. Combined with the FTIR and SEM results, the high-efficiency reinforcement may be due to the crosslinked structure of Si–O–Si hindering the hydrogen-bonded polyurethane segments but are beneficial to form organic and inorganic hybrid structures, resulting in increased hardness. Moreover, the increasing crosslinking density also plays an important role. In brief, increasing the MPTMS contents allows less penetration of the needle due to the increase in the crosslinking structure between chains as well as lower chain flexibility and facilitation of segment motion.

#### 3.2.5. Adhesive Property

**Figure 10:**
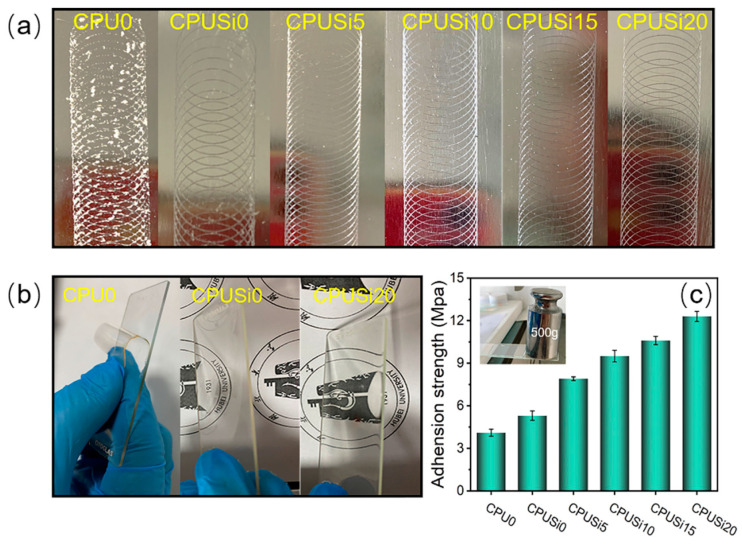
(**a**) Photos of the object behind the circle; (**b**) Photos of the object after boiling at high temperature; (**c**) The lap shear strength of CPU and CPUSi.

The adhesive property of coatings is enormously crucial because a poor adhesive will lead to failure of protection performance. As presented in Figure 10a, the sample without silane modification shows a terrible bond. By contrast, CPUSi exhibits much better adhesive property, significantly modified by IPDI trimer with mercaptosilane groups. From CPUSi5 to CPUSi20, the adhesive can reach grade 0 because the shedding area is almost 0. Figure 8b shows the adhesive property of CPU, CPUSi0, and CPUSi20 coatings after suffering three-hour high-temperature water cooking. It is convincing, however, that the adhesive property of the layer under mal-conditions is greatly improved by incorporating MPTMS groups. Just as in Figure 10b, the CPU0 coating had peeled off easily. Conversely, the CPUSi had been preserved intact. Incorporating MPTMS groups into the CPU structure played two roles in varying the adhesive property. On the one hand, Si–OH of hydrolyzed MPTMS reacts with the hydroxyl groups on the matrix surface to form a stable Si–O bond. On the other hand, the hydrophobic groups and the crosslinking structure hinder the erosion of the water molecules available. Thereby, CPUSi coating could show good adhesion, even in a hot and humid environment.

It is stated, therefore, that both the cohesive and adhesive strengths of the adhesives were relevant to the sample resistance to flow under shear, which could be expressed as the lap shear strengths. Moreover, the adhesion of the coating to the substrate was evaluated by the lap shear strengths according to GB/T 13936-92. As described in Figure 10c, the lap shear strength of CPUSi20 and CPUSi0 was 12.4 and 5.3 MPa, respectively, which is 302% and 129% higher than that of a neat CPU (4.1 MPa). Obviously, it can be said that the incorporation of MPTMS into the CPU increases the lap shear strength. This may be due to high-crosslinking from Si–O–Si units in CPUSi molecular chains, which decreases the mobility of the soft segment and increases the cohesion strength of CPUSi. What is more, it should be noted that there are many hydroxyl groups (–OH) in the substrate, so the hydrogen bonding and covalent bonding interactions between hydroxyl groups in the substrate surface and silicon hydroxyl groups (Si–OH) in the CPUSi play a role of great importance in the adhesion to the surface of the substrate.

#### 3.2.6. Thermal Insulation Performance of CPUSi/ATO

**Figure 11:**
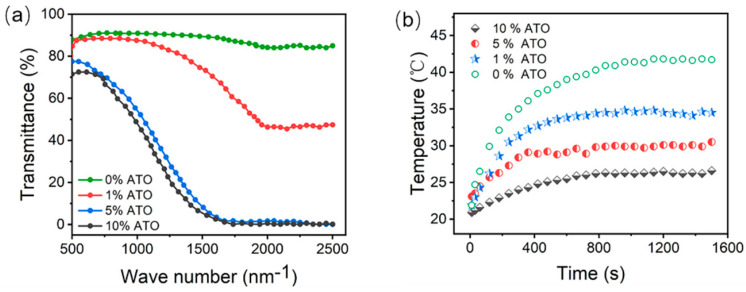
(**a**) The transmittance between 500 and 2500 band; (**b**) Thermal insulation curves of CPUSi/ATO composite coatings with different ATO contents.

The previous discussion found that CPUSi has a significant improvement in adhesion, strength, and weather resistance, which is obviously better than that of the bio-based polyurethane in other literature, and comparable to that of petroleum-based polyurethane (Supplementary Materials Table S1). Thus, we believe that CPUSi, as the base resin of thermal insulation coatings, has a potential application prospect. Here, we chose ATO nanoparticles as thermal insulation fillers and coated them on the glass plate to test their thermal insulation properties. It is well known that the infrared band (780–2500 nm) is the thermal band. Obviously, the transmittance of CPUSi/ATO dropped rapidly between this band after adding 1% ATO (Figure 11a), which can be attributed to the fact that ATO particles can suppress the spectral transmittance in the near-infrared region through the absorption mechanism. The infrared barrier rate is further improved by adding 5% and 10% ATO, but there is no significant difference between 5% and 10% ATO in the infrared barrier rate. Noteworthy, the increase of ATO also leads to decreased visible light transmittance (500–780 nm). Specifically, the transmittance of the composite coating of 1% ATO, 5% ATO, and 10% ATO is 88.4%, 71.5%, and 70.1%, respectively, which conforms to the GB7258-2017 and GB9656-2021.

Figure 11b shows the thermal insulation effect of CPUSi/ATO glass plates more intuitively in thermal insulation devices. Regardless of whether ATO is added or not, the extension of irradiation time will cause the temperature to rise rapidly until the temperature is basically stable. The temperature of CPUSi resin quickly rose to 41.7 °C in 25 min. After compounding with ATO, the temperature increases slowly, reaching equilibrium at 34.5 °C (1% ATO), 30.5 °C (5% ATO), and 26.6 °C (10% ATO), respectively, thus, the maximum temperature difference is 15.1°, which means that the CPUSi/ATO coating can effectively prevent heat transfer and thermal diffusion, and play an excellent thermal insulation effect.

## 4. Conclusions

In the present study, a novel isophorone diisocyanate with organosilicon modification was designed and then successfully synthesized with castor oil and poly (ethylene adipate) diol to prepare a series of organosilicon-containing bio-based polyurethanes (CPUSi). The molecular structure and morphologies and the optical performance, surface, thermal, mechanical properties, adhesive property, and chemical resistance properties of the hybrid films were characterized and measured. The results showed that IPDI-M contributed to forming a crosslinked network, which dissociated the hydrogen bonds and inhibited the aggregation of the hard segment. As the IPDI-M content increases, the optical performance of the hybrid coatings maintains high transmittance. Furthermore, the silica component significantly improved the mechanical properties and thermostability of the CPUSi film. The tensile strength, Young’s modulus, and hardness of the samples increased from 9.5 MPa to 22.3 MPa, 4.05 MPa to 81.59 MPa, and 0.03 GPa to 0.13 Gpa, respectively, while Td5 and T d50 increased from 288.2 °C and 352.7 °C to 294.8 °C and 381.5 °C, respectively. Moreover, the adhesive property, surface property, and chemical resistance properties of the CPU have been greatly enhanced from the introduction of MPTMS. Finally, in the application test of employing CPUSi as thermal insulation coating, CPUSi/ATO can effectively block the transmission of near-infrared rays. Compared with the glass-covered with CPUSi only, CPUSi/ATO can reduce the temperature by 15.1 °C.

## Figures and Tables

**Table 1:** The composition of raw material employed for the preparation of CPUSi.

Sample	IPDI-M/g(mol)		IPDI /g(mol)	CO/g (mol)	PEA /g(mol)	MPTMS /g(mol)
	IPDI-T	MPTMS				
CPU0	0	0	8.0 (0.072)	13.8 (0.015)	6 (0.02)	0
CPUSi0	0	0	8.0 (0.072)	13.8 (0.015)	6 (0.02)	2.36 (0.012)
CPUSi5	1.7 (0.0075)	0.5 (0.0025)	7.4 (0.067)	13.8 (0.015)	6 (0.02)	2.36 (0.012)
CPUSi10	3.3 (0.0150)	1.0 (0.0050)	6.9 (0.062)	13.8 (0.015)	6 (0.02)	2.36 (0.012)
CPUSi15	5.0 (0.0225)	1.5 (0.0075)	6.3 (0.057)	13.8 (0.015)	6 (0.02)	2.36 (0.012)
CPUSi20	6.7 (0.0300)	2.0 (0.0100)	5.8 (0.052)	13.8 (0.015)	6 (0.02)	2.36 (0.012)

**Table 2:** XPS peak characteristics of CPU0, CPUSi0, CPUSi10, and CPUSi20.

Sample	Atomic Concentration/%				Si Atomic Concentration/%	
	C	N	O	S	Theoretical Bulk	Experimental on the Surface
CPU0	57.63	0.21	42.16	0	0	0
CPUSi0	49.05	0.38	43.73	0.41	1.12	6.43
CPUSi10	37.34	0.43	48.13	0.6	1.44	13.5
CPUSi20	34.14	0.68	49.01	0.92	1.7	15.25

**Table 3:** Thermal parameters of the CPU and CPUSi films.

Samples	T_d5_/°C	T_d50_/°C	T_max1_/°C	T_max2_/°C	Residual Rate/%
CPU0	288.2	352.7	314.5	362.5	3.63
CPUSi0	304.1	362.3	333.8	387.0	5.15
CPUSi5	302.0	377	329.3	390.7	5.96
CPUSi10	301.2	378.8	327.3	391.5	6.24
CPUSi15	296.3	378.8	326.2	392.8	6.9
CPUSi20	294.8	381.5	330.5	393.3	7.4

## Data Availability

The authors declare data availability.

## Supplementary Materials

The following supporting information can be downloaded at: https://www.mdpi.com/article/10.3390/polym14091880/s1, Figure S1. 1H-NMR of IPDI-T (black line) and IPDI-M (red line); Table S1. Summary of the deeper comparison by other investigators and relevant studies of poly-urethane coatings. References [12,52,53,54,55,56,57,58,59] are cited in the Supplementary Materials.

## References

[1] Matsui H., Tabata H. (2021). Sn-Doped In2O3Nanoparticles as Thermal Insulating Materials for Solar–Thermal Shielding in the Infrared Range. ACS Appl. Nano Mater..

[2] Wang M., Bu J., Xu Y., Liu Y., Yang Y. (2021). Sb-doped SnO_2_ (ATO) hollow submicron spheres for solar heat insulation coating. Ceram. Int..

[3] Wu K., Xiang S., Zhi W., Bian R., Wang C., Cai D. (2017). Preparation and characterization of UV curable waterborne poly(urethane-acrylate)/antimony doped tin oxide thermal insulation coatings by sol-gel process. Prog. Org. Coat..

[4] Fang D., Yu H., Dirican M., Tian Y., Xie J., Jia D., Yan C., Liu Y., Li C., Liu H. (2021). Disintegrable, transparent and mechanically robust high-performance antimony tin oxide/nanocellulose/polyvinyl alcohol thermal insulation films. Carbohydr. Polym..

[5] Wang W., Liang Y., Yang Z., Zhang W., Wang S. (2019). Construction of ultraviolet protection, thermal insulation, superhydrophobic and aromatic textile with Al-doped ZnO–embedded lemon microcapsule coatings. Text. Res. J..

[6] Farshchi N., Gedan-Smolka M. (2020). Polyurethane Powder Coatings: A Review of Composition and Characterization. Ind. Eng. Chem. Res..

[7] Gao W.C., Wu W., Chen C.Z., Zhao H., Liu Y., Li Q., Huang C.X., Hu G.H., Wang S.F., Shi D. (2022). Design of a Superhydrophobic Strain Sensor with a Multilayer Structure for Human Motion Monitoring. ACS Appl Mater Interfaces.

[8] Zhao H., Gao W.C., Li Q., Khan M.R., Hu G.H., Liu Y., Wu W., Huang C.X., Li R.K.Y. (2022). Recent advances in superhydrophobic polyurethane: Preparations and applications. Adv. Colloid Interface Sci..

[9] Gunatillake P.A., Dandeniyage L.S., Adhikari R., Bown M., Shanks R., Adhikari B. (2018). Advancements in the Development of Biostable Polyurethanes. Polym. Rev..

[10] Phung Hai T.A., Tessman M., Neelakantan N., Samoylov A.A., Ito Y., Rajput B.S., Pourahmady N., Burkart M.D. (2021). Renewable Polyurethanes from Sustainable Biological Precursors. Biomacromolecules.

[11] Cuevas J.M., Seoane-Rivero R., Navarro R., Marcos-Fernandez A. (2020). Coumarins into Polyurethanes for Smart and Functional Materials. Polymers.

[12] Liang H., Li Y., Huang S., Huang K., Zeng X., Dong Q., Liu C., Feng P., Zhang C. (2019). Tailoring the Performance of Vegetable Oil-Based Waterborne Polyurethanes through Incorporation of Rigid Cyclic Rings into Soft Polymer Networks. ACS Sustain. Chem. Eng..

[13] Li C., Sung J., Sun X.S. (2015). Network from Dihydrocoumarin via Solvent-Free Metal-Mediated Pathway: A Potential Structure for Substantial Toughness Improvement of Epoxidized Plant Oil Materials. ACS Sustain. Chem. Eng..

[14] An X.-P., Chen J.-H., Li Y.-D., Zhu J., Zeng J.-B. (2018). Rational design of sustainable polyurethanes from castor oil: Towards simultaneous reinforcement and toughening. Sci. China Mater..

[15] Jian X.-Y., He Y., Li Y.-D., Wang M., Zeng J.-B. (2017). Curing of epoxidized soybean oil with crystalline oligomeric poly(butylene succinate) towards high performance and sustainable epoxy resins. Chem. Eng. J..

[16] Zhang C., Kessler M.R. (2015). Bio-based Polyurethane Foam Made from Compatible Blends of Vegetable-Oil-based Polyol and Petroleum-based Polyol. ACS Sustain. Chem. Eng..

[17] Sahoo S., Kalita H., Mohanty S., Nayak S.K. (2017). Meticulous study on curing kinetics of green polyurethane-clay nanocomposite adhesive derived from plant oil: Evaluation of decomposition activation energy using TGA analysis. J. Macromol. Sci. Phys. Part A.

[18] Heidarian M., Shishesaz M.R., Kassiriha S.M., Nematollahi M. (2010). Characterization of structure and corrosion resistivity of polyurethane/organoclay nanocomposite coatings prepared through an ultrasonication assisted process. Prog. Org. Coat..

[19] Hormaiztegui M.E.V., Daga B., Aranguren M.I., Mucci V. (2020). Bio-based waterborne polyurethanes reinforced with cellulose nanocrystals as coating films. Prog. Org. Coat..

[20] Pinto E.R.P., Barud H.S., Silva R.R., Palmieri M., Polito W.L., Calil V.L., Cremona M., Ribeiro S.J.L., Messaddeq Y. (2015). Transparent composites prepared from bacterial cellulose and castor oil based polyurethane as substrates for flexible OLEDs. J. Mater. Chem. C.

[21] Gurunathan T., Chung J.S. (2016). Physicochemical Properties of Amino–Silane-Terminated Vegetable Oil-Based Waterborne Polyurethane Nanocomposites. ACS Sustain. Chem. Eng..

[22] Akram D., Hakami O., Sharmin E., Ahmad S. (2017). Castor and Linseed oil polyurethane/TEOS hybrids as protective coatings: A synergistic approach utilising plant oil polyols, a sustainable resource. Prog. Org. Coat..

[23] Acuña P., Zhang J., Yin G.-Z., Liu X.-Q., Wang D.-Y. (2020). Bio-based rigid polyurethane foam from castor oil with excellent flame retardancy and high insulation capacity via cooperation with carbon-based materials. J. Mater. Sci..

[24] Lee J.H., Kim S.H. (2020). Fabrication of silane-grafted graphene oxide and its effect on the structural, thermal, mechanical, and hysteretic behavior of polyurethane. Sci. Rep..

[25] Zhang J., Zhang C., Song F., Shang Q., Hu Y., Jia P., Liu C., Hu L., Zhu G., Huang J. (2022). Castor-oil-based, robust, self-healing, shape memory, and reprocessable polymers enabled by dynamic hindered urea bonds and hydrogen bonds. Chem. Eng. J..

[26] Huo L., Wang D., Liu H., Jia P., Gao J. (2016). Cytoxicity, dynamic and thermal properties of bio-based rosin-epoxy resin/castor oil polyurethane/carbon nanotubes bio-nanocomposites. J. Biomater. Sci. Polym. Ed..

[27] Allauddin S., Narayan R., Raju K.V.S.N. (2013). Synthesis and Properties of Alkoxysilane Castor Oil and Their Polyurethane/Urea–Silica Hybrid Coating Films. ACS Sustain. Chem. Eng..

[28] Seeni Meera K.M., Murali Sankar R., Jaisankar S.N., Mandal A.B. (2013). Physicochemical studies on polyurethane/siloxane cross-linked films for hydrophobic surfaces by the sol-gel process. J. Phys. Chem. B.

[29] Wang L., Shen Y., Lai X., Li Z., Liu M. (2010). Synthesis and properties of crosslinked waterborne polyurethane. J. Polym. Res..

[30] Liang Z., Zhu J., Li F., Wu Z., Liu Y., Xiong D. (2021). Synthesis and properties of self-crosslinking waterborne polyurethane with side chain for water-based varnish. Prog. Org. Coat..

[31] Zhao H., Hao T.H., Hu G.H., Shi D., Huang D., Jiang T., Zhang Q.C. (2017). Preparation and Characterization of Polyurethanes with Cross-Linked Siloxane in the Side Chain by Sol-Gel Reactions. Materials.

[32] Fu C., Yang Z., Zheng Z., Shen L. (2014). Properties of alkoxysilane castor oil synthesized via thiol-ene and its polyurethane/siloxane hybrid coating films. Prog. Org. Coat..

[33] Guo S., Xu P., Yu H., Li X., Cheng Z. (2015). Hyper-branch sensing polymer batch self-assembled on resonant micro-cantilevers with a coupling-reaction route. Sens. Actuators B Chem..

[34] Shaik A., Narayan R., Raju K.V.S.N. (2014). Synthesis and properties of siloxane-crosslinked polyurethane-urea/silica hybrid films from castor oil. J. Coat. Technol. Res..

[35] Li Q., Guo L., Qiu T., Xiao W., Du D., Li X. (2016). Synthesis of waterborne polyurethane containing alkoxysilane side groups and the properties of the hybrid coating films. Appl. Surf. Sci..

[36] Li J.W., Lee H.T., Tsai H.A., Suen M.C., Chiu C.W. (2018). Synthesis and Properties of Novel Polyurethanes Containing Long-Segment Fluorinated Chain Extenders. Polymers.

[37] Gurunathan T., Chung J.S. (2017). Synthesis of aminosilane crosslinked cationomeric waterborne polyurethane nanocomposites and its physicochemical properties. Colloids Surf. A Physicochem. Eng. Asp..

[38] Jeon H.T., Jang M.K., Kim B.K., Kim K.H. (2007). Synthesis and characterizations of waterborne polyurethane–silica hybrids using sol–gel process. Colloids Surf. A Physicochem. Eng. Asp..

[39] Zhao H., Huang D., Hao T.-H., Hu G.-H., Ye G.-B., Jiang T., Zhang Q.-C. (2017). Synthesis and investigation of well-defined silane terminated and segmented waterborne hybrid polyurethanes. New J. Chem..

[40] Chattopadhyay D.K., Webster D.C. (2009). Thermal stability and flame retardancy of polyurethanes. Prog. Polym. Sci..

[41] Huang W., Huang J., Yu B., Meng Y., Cao X., Zhang Q., Wu W., Shi D., Jiang T., Li R.K.Y. (2021). Facile preparation of phosphorus containing hyperbranched polysiloxane grafted graphene oxide hybrid toward simultaneously enhanced flame retardancy and smoke suppression of thermoplastic polyurethane nanocomposites. Compos. Part A Appl. Sci. Manuf..

[42] Alagi P., Choi Y.J., Seog J., Hong S.C. (2016). Efficient and quantitative chemical transformation of vegetable oils to polyols through a thiol-ene reaction for thermoplastic polyurethanes. Ind. Crop. Prod..

[43] Dai M., Song P., Zhang Y. (2020). Preparation and characterization of modified castor oil via photo-click chemistry for UV-curable waterborne polyurethane with enhanced water resistance and low conductive percolation threshold. J. Appl. Polym. Sci..

[44] Ahn B.U., Lee S.K., Lee S.K., Park J.H., Kim B.K. (2008). UV curable polyurethane dispersions from polyisocyanate and organosilane. Prog. Org. Coat..

[45] Zhang J., Yao M., Chen J., Jiang Z., Ma Y. (2019). Synthesis and properties of polyurethane elastomers based on renewable castor oil polyols. J. Appl. Polym. Sci..

[46] Das S., Pandey P., Mohanty S., Nayak S.k. (2016). Effect of nanosilica on the physicochemical, morphological and curing characteristics of transesterified castor oil based polyurethane coatings. Prog. Org. Coat..

[47] Bockel S., Harling S., Grönquist P., Niemz P., Pichelin F., Weiland G., Konnerth J. (2020). Characterization of wood-adhesive bonds in wet conditions by means of nanoindentation and tensile shear strength. Eur. J. Wood Wood Prod..

[48] Gupta T.K., Singh B.P., Dhakate S.R., Singh V.N., Mathur R.B. (2013). Improved nanoindentation and microwave shielding properties of modified MWCNT reinforced polyurethane composites. J. Mater. Chem. A.

[49] Lin C., Ying P., Huang M., Zhang P., Yang T., Liu G., Wang T., Wu J., Levchenko V. (2021). Synthesis of robust and self-healing polyurethane/halloysite coating via in-situ polymerization. J. Polym. Res..

[50] Cai D., Jin J., Yusoh K., Rafiq R., Song M. (2012). High performance polyurethane/functionalized graphene nanocomposites with improved mechanical and thermal properties. Compos. Sci. Technol..

[51] Jindal P., Kumar N., Kumar D. (2017). Elastic Modulus Behavior of Multi-Walled Carbon Nano-Tubes/Polyurethane Composites using Nano-Indentation Techniques. Indian J. Sci. Technol..

[52] Shen R., Long M., Lei C., Dong L., Yu G., Tang J. (2022). Anticorrosive waterborne polyurethane coatings derived from castor oil and renewable diols. Chem. Eng. J..

[53] Cassales A., Ramos L.A., Frollini E. (2020). Synthesis of bio-based polyurethanes from Kraft lignin and castor oil with simultaneous film formation. Int. J. Biol. Macromol..

[54] Man L., Feng Y., Hu Y., Yuan T., Yang Z. (2019). A renewable and multifunctional eco-friendly coating from novel tung oil-based cationic waterborne polyurethane dispersions. J. Clean. Prod..

[55] Liang B., Zhao J., Li G., Huang Y., Yang Z., Yuan T. (2019). Facile synthesis and characterization of novel multi-functional bio-based acrylate prepolymers derived from tung oil and its application in UV-curable coatings. Ind. Crop. Prod..

[56] Li C., Xiao H., Wang X., Zhao T. (2018). Development of green waterborne UV-curable vegetable oil-based urethane acrylate pigment prints adhesive: Preparation and application. J. Clean. Prod..

[57] Zhang L., Kong Q., Kong F., Liu T., Qian H. (2019). Synthesis and surface properties of novel fluorinated polyurethane base on F-containing chain extender. Polym. Adv. Technol..

[58] Li K., Qi Y., Zhou Y., Sun X., Zhang Z. (2021). Microstructure and Properties of Poly(ethylene glycol)-Segmented Polyurethane Antifouling Coatings after Immersion in Seawater. Polymers.

[59] Lyu J., Xu K., Zhang N., Lu C., Zhang Q., Yu L., Feng F., Li X. (2019). In Situ Incorporation of Diamino Silane Group into Waterborne Polyurethane for Enhancing Surface Hydrophobicity of Coating. Molecules.

