# Supplementary material for: Synthesis and Characterization of Crosslinked Castor Oil-Based Polyurethane Nanocomposites Based on Novel Silane-Modified Isocyanate and Their Potential Application in Heat Insulating Coating
Source: Polymers (Basel). 2022 May 4;14(9):1880. doi: 10.3390/polym14091880 (PMC9105965; doi:10.3390/polym14091880)
Supplement: Supplementary file 1 [file polymers-14-01880-s001.zip › polymers-1680324-supplementary.pdf]

polymers
MDPI

Supplementary Materials

Synthesis and Characterization of Crosslinked Castor Oil-Based Polyurethane Nanocomposites Based on Novel Silane-Modified Isocyanate and Their Potential Application in Heat Insulating Coating

Yuan Meng [1,2], Ken Chen [1], Yuyin Yang [1], Tao Jiang [1], Tonghui Hao [1], Xiaoju Lu [2,*] and Qunchao Zhang [1,*]

[1] School of Materials Science and Engineering, Hubei University, Wuhan 430000, China; 201901111300099@stu.hubu.edu.cn (Y.M.); 202121113012842@stu.hubu.cn (K.C.); 202121113012884@stu.hubu.edu.cn (Y.Y.); jiangtao@hubu.edu.cn (T.J.); haoth@hubu.edu.cn (T.H.)
[2] School of Chemistry and Chemical Engineering, Hubei Polytechnic University, Huangshi 430000, China
* Correspondence: luxiaoju@hbpu.edu.cn (X.L.); zhangqc1976@hubu.edu.cn (Q.Z.)

Table S1. Summary of the deeper comparison by other investigators and relevant studies of polyurethane coatings.

	Water contact angle	swelling ratio	Adhesion	Solvent Resistance	Solvent
CPUSi	88.5	0.12% (H_2O); 68.16% (DMF)	12.4MPa	0.13% (H_2SO_4); 1.28% (NaOH); 1.47% (EAc); 0.42% (CP); 7.19% (DCP)	Butanone
PU based on isosorbide and L-tyosine [52]	92.8	N/A	N/A	N/A	Water
PU based on kraft and CO [53]	74	70% (DMF)	N/A	N/A	Solvent free
PU based on OPD and CO [12]	72	N/A	4B	N/A	Water
PU based on tund oil [54]	100	4.9% (H_2O)	N/A	N/A	Water
PU based on tund oil [55]	N/A	1.7% (H_2O)	N/A	N/A	Acetone
PU based on PCL [56]	91.04	8.49% (H_2O)	N/A	N/A	Water
PU based on PTMG [57]	105	1.36% (H_2O)	0	N/A	ethyl acetate
PU based on PEG and PPG [58]	85	153% (H_2O)	N/A	N/A	N/A
PU based on APTS and PCDL [59]	86	3.9% (H_2O)	N/A	N/A	Water

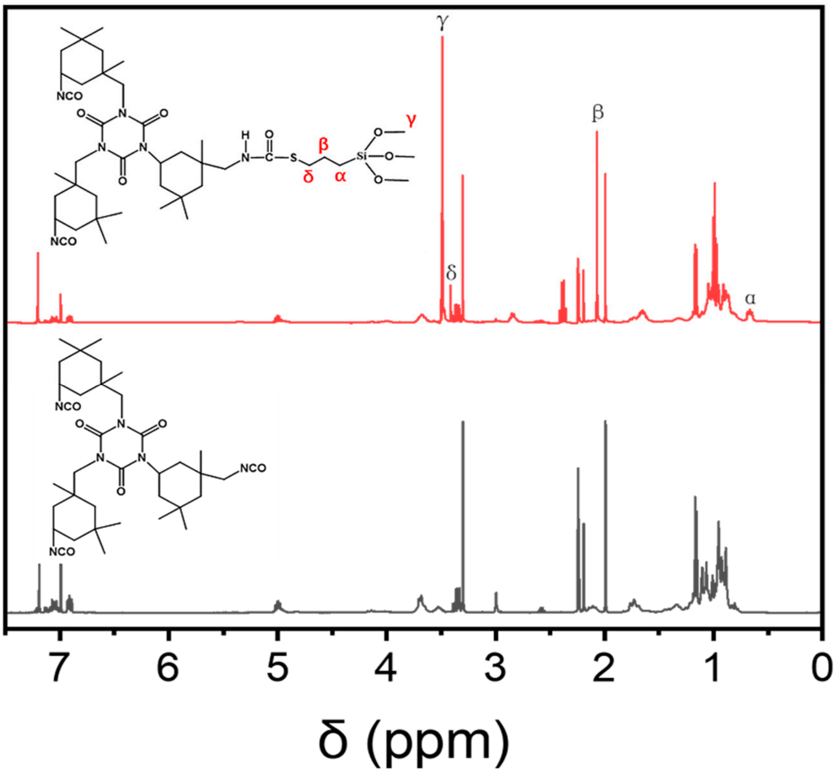

Figure S1. ^{1}H-NMR of IPDI-T (black line) and IPDI-M (red line).

1. Shen, R.; Long, M.; Lei, C.; Dong, L.; Yu, G.; Tang, J. Anticorrosive waterborne polyurethane coatings derived from castor oil and renewable diols. *Chem. Eng. J.* **2022**, *433*, doi:10.1016/j.cej.2021.134470.
2. Cassales, A.; Ramos, L.A.; Frollini, E. Synthesis of bio-based polyurethanes from Kraft lignin and castor oil with simultaneous film formation. *Int. J. Biol. Macromol.* **2020**, *145*, 28-41, doi:10.1016/j.ijbiomac.2019.12.173.
3. Liang, H.; Li, Y.; Huang, S.; Huang, K.; Zeng, X.; Dong, Q.; Liu, C.; Feng, P.; Zhang, C. Tailoring the Performance of Vegetable Oil-Based Waterborne Polyurethanes through Incorporation of Rigid Cyclic Rings into Soft Polymer Networks. *ACS Sustain. Chem. Eng.* **2019**, *8*, 914-925, doi:10.1021/acssuschemeng.9b05477.
4. Man, L.; Feng, Y.; Hu, Y.; Yuan, T.; Yang, Z. A renewable and multifunctional eco-friendly coating from novel tung oil-based cationic waterborne polyurethane dispersions. *Journal of Cleaner Production* **2019**, *241*, doi:10.1016/j.jclepro.2019.118341.
5. Liang, B.; Zhao, J.; Li, G.; Huang, Y.; Yang, Z.; Yuan, T. Facile synthesis and characterization of novel multi-functional bio-based acrylate prepolymers derived from tung oil and its application in UV-curable coatings. *Industrial Crops and Products* **2019**, *138*, doi:10.1016/j.indcrop.2019.111585.
6. Li, C.; Xiao, H.; Wang, X.; Zhao, T. Development of green waterborne UV-curable vegetable oil-based urethane acrylate pigment prints adhesive: Preparation and application. *Journal of Cleaner Production* **2018**, *180*, 272-279, doi:10.1016/j.jclepro.2018.01.193.
7. Zhang, L.; Kong, Q.; Kong, F.; Liu, T.; Qian, H. Synthesis and surface properties of novel fluorinated polyurethane base on F‐containing chain extender. *Polym. Adv. Technol.* **2019**, *31*, 616-629, doi:10.1002/pat.4802.
8. Li, K.; Qi, Y.; Zhou, Y.; Sun, X.; Zhang, Z. Microstructure and Properties of Poly(ethylene glycol)-Segmented Polyurethane Antifouling Coatings after Immersion in Seawater. *Polymers (Basel)* **2021**, *13*, doi:10.3390/polym13040573.
9. Lyu, J.; Xu, K.; Zhang, N.; Lu, C.; Zhang, Q.; Yu, L.; Feng, F.; Li, X. In Situ Incorporation of Diamino Silane Group into Waterborne Polyurethane for Enhancing Surface Hydrophobicity of Coating. *Molecules* **2019**, *24*, doi:10.3390/molecules24091667.
